# Nuclear gene phylogeography using PHASE: dealing with unresolved genotypes, lost alleles, and systematic bias in parameter estimation

**DOI:** 10.1186/1471-2148-10-118

**Published:** 2010-04-30

**Authors:** Ryan C Garrick, Paul Sunnucks, Rodney J Dyer

**Affiliations:** 1 Department of Biology, Virginia Commonwealth University, Richmond, Virginia 23284, USA; 2 Current Address: Department of Ecology & Evolutionary Biology, Yale University, New Haven, Connecticut 06520, USA; 3 Australian Centre for Biodiversity, School of Biological Sciences, Monash University, Clayton, Victoria 3800, Australia

## Abstract

**Background:**

A widely-used approach for screening nuclear DNA markers is to obtain sequence data and use bioinformatic algorithms to estimate which two alleles are present in heterozygous individuals. It is common practice to omit unresolved genotypes from downstream analyses, but the implications of this have not been investigated. We evaluated the haplotype reconstruction method implemented by PHASE in the context of phylogeographic applications. Empirical sequence datasets from five non-coding nuclear loci with gametic phase ascribed by molecular approaches were coupled with simulated datasets to investigate three key issues: (1) haplotype reconstruction error rates and the nature of inference errors, (2) dataset features and genotypic configurations that drive haplotype reconstruction uncertainty, and (3) impacts of omitting unresolved genotypes on levels of observed phylogenetic diversity and the accuracy of downstream phylogeographic analyses.

**Results:**

We found that PHASE usually had very low false-positives (i.e., a low rate of confidently inferring haplotype pairs that were incorrect). The majority of genotypes that could not be resolved with high confidence included an allele occurring only once in a dataset, and genotypic configurations involving two low-frequency alleles were disproportionately represented in the pool of unresolved genotypes. The standard practice of omitting unresolved genotypes from downstream analyses can lead to considerable reductions in overall phylogenetic diversity that is skewed towards the loss of alleles with larger-than-average pairwise sequence divergences, and in turn, this causes systematic bias in estimates of important population genetic parameters.

**Conclusions:**

A combination of experimental and computational approaches for resolving phase of segregating sites in phylogeographic applications is essential. We outline practical approaches to mitigating potential impacts of computational haplotype reconstruction on phylogeographic inferences. With targeted application of laboratory procedures that enable unambiguous phase determination via physical isolation of alleles from diploid PCR products, relatively little investment of time and effort is needed to overcome the observed biases.

## Background

The increasing use of nuclear DNA (nDNA) sequences in phylogeographic studies, in combination with sequence data from a haploid organellar locus, has been driven by the considerable improvements in strength and accuracy of historical inference that multi-locus analyses can provide [1]. The development of conserved intron-spanning polymerase chain reaction (PCR) primers has facilitated amplification of low- or single-copy nuclear loci in non-model species [2,3], and anonymous nuclear sequence loci have also been successfully applied in phylogeographic studies of diverse taxa (e.g., arthropods [4,5]; reptiles [6]; birds [7]). However, assaying nDNA sequence variation for reasonably large population-genetic sample sizes remains a major challenge [8]. There are several molecular laboratory techniques suitable for screening codominant markers by physically isolating sequence-variable alleles [9], but none have been broadly adopted owing to perceived or real logistical and/or technical limitations (e.g., high cost and time commitment, need for specialist equipment, difficulty resolving new or weakly amplifying alleles, and susceptibility to artefacts such as PCR recombination).

Algorithm-driven reconstruction of nuclear allele haplotypes following direct sequencing of diploid PCR products has become increasingly popular in phylogeographic and related applications (Additional file 1). These methods are based on the premise that the phase of alleles occurring in either homozygotes or heterozygotes that are polymorphic at only a single position can be resolved without ambiguity, and so this information can assist in resolving the phase of multi-site heterozygotes. One major advantage is that the per-locus cost of population screening is comparable to sequencing a fragment of animal mitochondrial DNA or plant chloroplast DNA, which is now quite routine and for which the necessary equipment and expertise are usually readily available. In addition, resolving power is thought to be quite good (all single nucleotide polymorphisms in a heterozygous genotype should be detectable). At present, one of the most widely used haplotype reconstruction methods is implemented in the software PHASE [10,11]. This Bayesian approach employs a neutral coalescent prior, making it suitable for population-genetic datasets, and it is able to accommodate recombination. Moreover, because PHASE uses Markov Chain Monte Carlo to sample the posterior distribution of potential haplotype pairs that could account for an observed ambiguous genotype, confidence probabilities for the phase of each segregating site and for each reconstructed haplotype pair can be estimated. In the context of genotype-phenotype association studies, PHASE has been shown to perform quite well with simulated and/or empirical human genetic datasets, but it is also frequently reported that rare haplotypes are susceptible to inference error [10-18]. This suggests that in applications where rare haplotypes are informative, computational approaches alone may be inadequate [19].

To date, few assessments of PHASE have been performed using non-coding gene regions, or datasets from species with complex evolutionary histories that are typical of non-human phylogeographic studies. However, patterns of variation at nuclear loci may be impacted by features of organismal biology such as effective population size, or past events and processes including population fragmentation, long-term isolation in refugia, and/or the existence of semipermeable landscape-level barriers to gene flow. For example, nuclear gene phylogeography of arthropods has revealed that extant taxa can exhibit highly polymorphic loci with many alleles segregating in large, deeply subdivided populations [4,5,20,21], and hybridization at zones of secondary contact can potentially generate genotypes comprised of distantly-related or novel recombinant allele haplotypes [22]. Previously, Huang *et al*. [23] performed the first assessment of PHASE using a large population-genetic dataset from a non-model species (526 individuals of the migratory locust, *Locusta migratoria*). The anonymous single copy nuclear locus examined in that study was characterized by high overall heterozygosity (*H*_O _= 0.66) and many rare alleles, with 87.8% of the 115 distinct multi-site heterozygote genotypes present at frequencies <1%. The authors reported that 9% of individuals with ambiguous genotypes remained unresolved owing to confidence probability values below the chosen PHASE threshold of 0.95. In a smaller scale study, Harrigan *et al*. [24] examined PHASE performance using a sample of 30 dabbling duck (*Anas *spp.) individuals with ambiguous genotypes, and found that 13-16.7% were unresolved when running the software with comparable settings.

Broadly speaking, genetic datasets known to be impacted by technical artefacts that operate in a non-random manner need to be analyzed and interpreted with caution (e.g., non-amplifying 'null' alleles [25]). However, an examination of papers focusing on phylogeography, hybridization and speciation indicates that in most empirical applications of PHASE, unresolved genotypes are simply omitted from the dataset (Table 1). Although this practice is generally considered to have negligible impacts on subsequent estimates of population genetic parameters and associated phylogeographic inferences [26], no formal assessment has been performed to date [27]. Indeed, there are reasons to believe that the duel loss of rare alleles and heterozygous genotypes could introduce systematic bias into downstream analyses. For example, consider the parameter Θ (4*N*_*e*_μ for diploid autosomal genes, where *N*_*e *_is effective population size and μ is the locus-specific per-generation mutation rate). This parameter is central to widely-used coalescent phylogeographic analyses (e.g., population size changes [28]; migration matrix estimation [29]; isolation-with-migration divergence modelling [30]), and *N*_*e*_-values derived from Θ underpin simulation-based tests of alternative vicariance scenarios [4,21,31-33]. The estimation of Θ is heavily dependent on the number of segregating sites in a sample of sequences [34], and rare alleles usually contribute new segregating sites. It is therefore possible that computational haplotype reconstruction leads to the removal of a sufficiently large number of genotypes containing rare alleles so as to downwardly bias Θ. The potential for systematic bias also extends to other analyses. For example, contrasts between within-population heterozygosity and the number of alleles form the basis of tests for recent genetic bottlenecks [35], and the frequency distributions of allele haplotypes [36] or pairwise nucleotide differences [37] are commonly used to distinguish exponential growth from population size constancy (Table 1).

**Table 1:** Literature survey of empirical studies focusing on phylogeography, hybridization and speciation that used PHASE for haplotype reconstruction (see Additional file 4 for a complete list of references).

	Birds	Herpetofauna	Mammals	Fish	Invertebrates	Total
	(*N *= 15)	(*N *= 12)	(*N *= 10)	(*N *= 8)	(*N *= 15)	(*N *= 60)
**PHASE threshold used**						
0.95	3	-	1	4	2	*10*
0.90	-	4	1	1	2	*8*
0.75-0.80	4	-	-	-	-	*4*
0.70	1	1	-	-	-	*2*
0.60	-	-	-	2	3	*5*
Best of replicate runs^a^	-	1	-	-	2	*3*
Not reported	7	6	8	1	6	*28*

**Unresolved genotypes**						
Excluded or coded as missing	5	5	1	5	3	*19*
Resolved experimentally	2	-	-	-	2	*4*
Included despite uncertainty	3	-	-	-	-	*3*
None present	1	1	-	3	4	*9*
Not reported	4	6	9	-	6	*25*

**Experimental validation**						
Cloning	1	2	-	1	4	*8*
Allele-specific PCR	3	-	-	-	-	*3*
None	11	10	10	7	11	*49*

**Downstream analyses**						
Theta (Θ)^b^	14	6	9	4	7	*40*
Nucleotide diversity (Π)	9	7	9	4	7	*36*
Neutrality or population growth	7	6	4	6	5	*28*
Network or phylogenetic tree	10	9	9	6	10	*44*

^a^Uses all inferred haplotypes from the run with the best average goodness-of-fit

^b^Includes studies that implicitly calculated theta as part of coalescent analyses (e.g., MIGRATE, FLUCTUATE, IM)

In the present paper we assess the performance of PHASE using five large nDNA sequence datasets from two Collembola species (Hexapoda), for which all genotypes have been resolved by laboratory procedures [38]. In addition, we analyze 35 simulated datasets with contrasting levels of polymorphism and, for the first time, examine the impact of unresolved genotypes and 'lost alleles' on downstream phylogeographic analyses. Outcomes are considered under PHASE confidence probability thresholds of 0.90 (i.e., the default value used by the software) and 0.60, both of which are commonly used in the relevant empirical literature (Table 1). We conclude with a discussion of the complementarity of laboratory-based physical isolation of alleles and computational haplotype reconstruction.

## Methods

### Literature survey

Papers citing Stephens *et al*. [10] or Stephens and Donnelly [11], and focusing on phylogeography, speciation or hybridization of natural populations of non-primate animals, were identified using Web of Science^® ^(accessed December 2009). Literature searches were conducted using the keywords "phylogeograph*", "speciation", or "gene flow" to find relevant papers in any journal, and by examining all citing articles in *BMC Evolutionary Biology*, *Evolution*, *Molecular Ecology*, and *Molecular Phylogenetics and Evolution*. If papers were within the scope of this survey, the Methods and Results sections were examined. Papers that primarily used PHASE in conjunction with non-coding nDNA sequence data were included, because we wanted to determine how the software was being used, and the types of downstream analyses that were performed using computationally-phased datasets.

### Datasets and polymorphism levels

Our empirical nDNA sequence datasets were generated as part of a comparative phylogeographic investigation that included two saproxylic Collembola species [5,39,40]. Sequence variation at five nuclear loci was assayed in >200 individuals of either *Acanthanura *sp. nov. (three loci, prefix '*Uc*') or Pseudachorutinae gen. nov. sp. nov. (two loci, prefix '*Sm*'; Table 2). These markers included an intron (*elongation factor-1α*; *EF1α*) and three non-coding anonymous loci. The number of nucleotides ranged from 92-266-bp, and alleles were phased by physically isolating them from diploid PCR products using single-stranded conformation polymorphism (SSCP) followed by targeted DNA sequencing [38,41]. This experimental approach minimizes artefacts that can arise from PCR recombination because it is cloning-free. Marker development and population screening methods are given in Garrick and Sunnucks [38]. Four of the five nuclear loci had alleles with several discontiguous insertion/deletion (indel) mutations. These were recoded using arbitrary nucleotide characters, with contiguous multi-base indels treated as a single event. In the present study, a 28-bp region of locus *Sm2 *(positions 138-165) was removed owing to unusually high polymorphism that exceeded the limits of PHASE. To reconstruct the sequences that would have been generated by direct sequencing of diploid PCR products from these five loci (i.e., with ambiguity codes), the two alleles from an individual genotype were collapsed into a consensus sequence using MESQUITE v2.5 [42]. Because this transformation of phase-known to ambiguous data includes no scoring error, our datasets represent idealized conditions.

**Table 2:** Characteristics of five empirical and 35 simulated datasets used in the present study.

Empirical		Ambiguous	Polymorphism measure				Simulated		Ambiguous	Polymorphism measure			
datasets	*N*	genotypes	*S*	*A*_N_	*G*_N_	*H*_O_	datasets	*N*	genotypes	*S*	*A*_N_	*G*_N_	*H*_O_
Pseudachorutinae sp.							Sim01	50	5	5	6	6	0.26
							
*Sm2 *Pop1	80	0	2	3	4	0.03	Sim02	50	14	5	6	11	0.58
*Sm2 *Pop2	61	0	2	3	3	0.02	Sim03	50	2	5	6	9	0.66
*Sm2 *Pop3	118	2	9	5	7	0.14	Sim04	50	9	5	6	9	0.50
*Sm2 *Pop4	62	0	2	4	4	0.03	Sim05	50	6	5	6	8	0.50
*Average*	*80*	*1*	*4*	*4*	*5*	*0.06*	Sim06	50	25	10	8	12	0.76
							
*SmEF1α *Pop1	81	2	9	6	9	0.10	Sim07	50	34	10	9	17	0.90
*SmEF1α *Pop2	60	8	6	3	4	0.13	Sim08	50	25	10	10	22	0.76
*SmEF1α *Pop3	105	40	11	9	12	0.39	Sim09	50	38	10	9	15	0.80
*SmEF1α *Pop4	54	9	6	6	8	0.22	Sim10	50	31	10	9	23	0.80
*Average*	*75*	*15*	*8*	*6*	*8*	*0.21*	Sim11	50	36	15	11	17	0.82
							
*Acanthanura *sp.							Sim12	50	38	15	12	30	0.86
							
*Uc3 *Pop1	26	0	2	3	4	0.08	Sim13	50	31	15	10	20	0.78
*Uc3 *Pop2	19	0	0	1	1	0.00	Sim14	50	32	15	11	19	0.76
*Uc3 *Pop3	67	3	8	8	9	0.24	Sim15	50	36	15	12	23	0.80
*Uc3 *Pop4	78	6	7	7	12	0.14	Sim16	50	39	20	13	31	0.90
*Uc3 *Pop5	15	8	17	9	11	0.67	Sim17	50	30	20	16	33	0.72
*Average*	*41*	*3*	*7*	*6*	*7*	*0.23*	Sim18	50	24	20	13	22	0.62
							
*Uc180 *Pop1	24	5	7	5	8	0.54	Sim19	50	27	20	12	21	0.62
*Uc180 *Pop2	19	1	2	2	2	0.05	Sim20	50	41	20	15	30	0.86
*Uc180 *Pop3	67	0	2	3	3	0.03	Sim21	50	40	25	19	37	0.90
*Uc180 *Pop4	78	0	1	2	2	0.01	Sim22	50	32	25	15	22	0.70
*Uc180 *Pop5	15	0	0	1	1	0.00	Sim23	50	30	25	17	32	0.72
*Average*	*41*	*1*	*2*	*3*	*3*	*0.13*	Sim24	50	20	25	13	21	0.70
							
*UcEF1α *Pop1	26	6	6	6	8	0.23	Sim25	50	35	25	18	29	0.84
*UcEF1α *Pop2	19	1	7	4	4	0.16	Sim26	50	40	30	20	39	0.84
*UcEF1α *Pop3	67	21	15	10	11	0.36	Sim27	50	41	30	17	36	0.90
*UcEF1α *Pop4	78	12	14	10	11	0.26	Sim28	50	32	30	19	29	0.82
*UcEF1α *Pop5	15	0	0	1	1	0.00	Sim29	50	40	30	21	38	0.90
*Average*	*41*	*8*	*8*	*6*	*7*	*0.20*	Sim30	50	41	30	22	42	0.92
							
*Sm2*	321	2	13	11	14	0.07	Sim31	50	37	35	17	35	0.88
*SmEF1α*	300	59	17	17	27	0.24	Sim32	50	41	35	17	36	0.94
*Uc3*	205	17	26	17	30	0.19	Sim33	50	41	35	16	32	0.88
*Uc180*	203	6	12	9	13	0.08	Sim34	50	46	35	20	38	0.92
*UcEF1α*	205	40	27	21	32	0.26	Sim35	50	38	35	15	28	0.80
*Average*	*247*	*25*	*19*	*15*	*23*	*0.17*	*Average*	*50*	*31*	*20*	*13*	*25*	*0.77*

Genetically distinct populations of two Collembola species identified previously [5,40] were pooled prior to reanalysis using PHASE, but are separated here for comparison with the single-population simulated datasets. *N *is the number of diploid individuals, and 'ambiguous genotypes' are those containing at least two heterozygous sites. Polymorphism measures are: *S*, number of segregating sites; *A*_N_, number of different alleles; *G*_N_, number of different genotypes; *H*_O_, observed heterozygosity.

Simulated DNA sequence datasets comprising 50 diploid genotypes sampled from a hypothetical panmictic population of constant size were generated with MESQUITE. Given that it is common for screening of nDNA loci to be performed on a subset of individuals taken from a larger phylogeographic study sample (e.g., 28 of the 60 studies included in our literature survey had *total *sample sizes of ≤ 100 individuals per nDNA locus per species), our chosen sample size achieves a balance between statistical power and the reality of empirical datasets. Coalescent gene trees with 100 allele copies (i.e., terminal branches) were simulated backward-in-time within an isolated population of haploid *N*_*e *_= 1,000 individuals and age = 2,000 organismal generations. This scheme of 2*N*_*e *_generations since isolation represents the theoretical expectation of the average time taken for alleles at a haploid locus to become reciprocally monophyletic on a gene tree. We did not use more complex models that included growth or decline because we wanted to assess PHASE performance under a best-case scenario, where the underlying assumptions of neutral coalescence within a single unstructured population of constant size were satisfied. Next, nucleotide characters (250-bp) were evolved forward-in-time along the branches of the coalescent gene trees using a HKY85 substitution model (root states and equilibrium base frequencies: A 0.30, C 0.20, G 0.15, T 0.35; ts/tv = 2.5). This substitution model and base frequency set is representative of our empirical Collembola data, and also typical of nDNA loci assayed for other organisms, as reported in the phylogeographic literature. To ensure independence, only one DNA sequence dataset was simulated per coalescent tree. During this process, scaling factors were used to convert branch lengths of coalescent gene trees (measured in organismal generations) into units that are typical of DNA sequence datasets (e.g., substitutions per site). To obtain levels of polymorphism that span the full range seen in empirical studies, scaling factors were determined by trial-and-error. We used the number of different alleles (*A*_N_) and segregating sites (*S*), calculated using DNASP v4.10.3 [43], as yardsticks of overall polymorphism. Scaling factor values between 1.0 × 10^-5 ^to 9.0 × 10^-6 ^generated a pool of 500 datasets, and from these we arbitrarily selected 35 datasets with *S*-values of 5, 10, 15, 20, 25, 30 or 35 (five datasets each; Table 2; Additional file 2). Within each of these 35 datasets, diploid genotypes were manually constructed by randomly pairing two haploid DNA sequences (i.e., alleles), to generate a diploid genotype. This represented random mating in a sexual out-crossing species. To mimic the phase-unknown genotypes produced from direct sequencing of diploid PCR products, consensus sequences for each genotype, with standard IUPAC nucleotide ambiguity codes at heterozygous positions, were generated in MESQUITE (as for the empirical Collembola data, above). In addition to calculating *S *and *A*_N _for each empirical and simulated dataset, we also quantified overall polymorphism via the number of different genotypes (*G*_N_), and observed heterozygosity (*H*_O_). Although the four summary statistics are non-independent (Additional file 3), they do reflect different components of the standing genetic variation, and so it is useful to investigate their relationships with PHASE performance.

### PHASE error, unresolved genotypes and lost alleles

Simulated and empirical datasets were analyzed using PHASE v2.1.1 [10,11], with files formatted in SeqPHASE [44]. We employed the MR model which makes explicit allowance for intragenic recombination, and compared to the non-recombination model, it performs better. However, the primary reason for choosing this model was because assessing evidence for recombinant alleles (c.f. making *a priori *assumptions about their absence) is important when using nDNA sequences in empirical population-level studies. For tri-allelic single nucleotide polymorphisms, the parent-independent mutation model was used. Runs consisted of 500 iterations as burn-in, 500 main iterations, and thinning interval = 1. Datasets were run three times with a different starting seed, and consistency across runs was checked by eye. The replicate with the best average goodness-of-fit value was used in subsequent analyses.

In the present paper we focus on the accuracy of PHASE in reconstructing whole haplotypes (c.f. individual single nucleotide polymorphisms) and diploid genotypes under two alternative thresholds, 0.60 and 0.90. These values encompass commonly used cut-offs (Table 1; note that the PHASE default is 0.90, and if not explicitly reported by studies included in the literature survey, we assumed the default value was used). Under each threshold, three aspects of performance were investigated:

*N*_ERR _Number of *individuals *with a confidently resolved (above-threshold) haplotype pair that included errors.

*N*_LCP _Number of *individuals *with genotypes that remained unresolved due to low confidence probability values (i.e., below-threshold). We also quantified the number of these individuals with correctly and incorrectly inferred haplotype pairs (*N*_LCP correct _and *N*_LCP incorrect _respectively).

*N*_LOST _Number *distinct gene lineages *(i.e., different alleles) that were present in the original phase-known dataset but were lost as a consequence of excluding individuals with unresolved (*N*_LCP_) genotypes.

#### Error

There have been contrasting reports regarding the relationship between PHASE error and the number of heterozygous sites in an ambiguous genotype (i.e., negative [14]; positive [18]; no relationship [23]), so we assessed the correlation between the latter and the sum of *N*_ERR _+ *N*_LCP incorrect_. We also examined which type of mistake was most prevalent in cases where PHASE haplotype pair error is analogous to false positives (*N*_ERR_), as well for those where the software appropriately indicated low confidence probability values (*N*_LCP incorrect_), using the following four categories that capture all observed mistakes:

*E*_COR + NOV _One haplotype is correct, the other is novel (did not previously exist).

*E*_COR + MIS _One haplotype is correct, the other is misidentified (previously existing).

*E*_MIS + MIS _Both haplotypes are misidentified.

*E*_MIS + NOV _One haplotype is misidentified, the other is novel.

#### Unresolved genotypes

Intuitively, phase determination for highly variable nuclear sequence loci should be more challenging than for relatively invariable markers. Despite this expectation, there is still a relatively limited understanding of the specific features of a dataset that drive uncertainty associated with haplotype pair reconstruction (i.e., which particular aspects of genetic variation), and so this warrants further investigation. Similarly, although the presence of rare alleles is known to contribute to difficulties with inferring phase of segregating sites in multi-site heterozygotes, there is little information on the importance of the specific genotypic configurations in which rare alleles occur. First, to determine whether relationships between *N*_LCP _and each of the four measures of dataset polymorphism levels (*S*, *A*_N_, *G*_N _and *H*_O_) exhibit different relative strengths, regression analyses were performed, with comparisons made using *R*^2^-values. Second, for each unresolved genotype in our simulated datasets, population frequencies of the two constituent alleles were calculated. These data were then summarized in box plots to assess the prevalence of small versus large asymmetries (e.g., pairing of two rare alleles, c.f. pairing of a common and a rare allele). Corresponding plots were constructed for empirical datasets.

#### Lost alleles

We employed two analytical approaches to assess the impact of omitting unresolved (*N*_LCP_) genotypes on 'phylogenetic diversity' [45]. To examine overall loss of distinct gene lineages, *N*_LOST _values were scaled by the number of different alleles in each original dataset, and a regression analysis was performed using corresponding *N*_LCP _values as the predictor variable. We also investigated whether lost alleles tended to be more divergent than other alleles in the dataset, as measured by proportion of nucleotide differences between a pair of sequences (uncorrected *p*) calculated in MEGA v4.0 [46]. We chose this simple measure of genetic distance (c.f. HKY-corrected distances) because the vast majority of polymorphic sites (97%) were consistent with an infinite-alleles mutation model, and uncorrected *p *is often used for nDNA sequence datasets in population-level studies. We plotted the difference between the mean from only those pair-wise comparisons involving the lost allele under consideration (*p*_LOST_) and the mean of all pair-wise comparisons within a dataset (*p*_DATASET_). Datasets with >1 lost allele have non-independent data points because multiple comparisons are made using the same *p*_DATASET _value. In these cases (24 simulated and one empirical dataset), *p*_LOST _values were first summed, and then deducted from *p*_DATASET_, thereby generating a single data point per dataset.

### Standard phylogeographic analyses

The omission of unresolved genotypes from a genetic dataset could potentially introduce biases into downstream phylogeographic analyses. We assessed the magnitude and directionality of differences in point estimates of two measures of population genetic diversity (Watterson's [34] Θ_W_, and Nei's [47] π), as well as two measures of demographic growth or neutrality (Tajima's [48]*D *and Fu's [36]*F*_S_). Empirical studies focusing on phylogeography, hybridization and speciation that use PHASE often estimate these parameters (Table 1). For each simulated dataset, parameter values were calculated in DNASP for the original phase-known dataset, and then recalculated after removing unresolved genotypes under each of the two thresholds. Significance of differences in parameter values was assessed via one-tailed paired t-tests, implemented in STATSDIRECT v2.7.7 http://www.statsdirect.com. Finally, we investigated the extent to which lost alleles can alter estimated root probabilities in intraspecific haplotype networks. To do this, statistical parsimony networks constructed for simulated datasets using TCS v1.21 [49] with the 95% confidence criterion enforced. The allele with the highest outgroup weight was determined for each of the original phase-known datasets, and then compared to results obtained when constructing statistical parsimony networks for the corresponding 'pruned' (90-*N*_LCP _and 60-*N*_LCP_) datasets.

## Results

### Literature survey

Sixty papers from 18 journals met our search criteria (Table 1 and Additional file 4). Each major vertebrate group and a diversity of invertebrates were represented. Based on those studies that reported how unresolved genotypes were treated, the most common course of action is complete exclusion. In some cases, the overall reduction in dataset size per locus was considerable (e.g., 21.9% [50], 13.6% [51], up to 8.5% [52]). Few studies used laboratory procedures in conjunction with computational phasing. Although the 60 papers used a variety of population-level analyses, Θ and π were frequently estimated. Many studies also examined evidence for selection acting on nDNA and/or demographic growth using Fu's *F*_S_, Tajima's *D*, mismatch distributions or related statistical procedures, and phylogenetic relationships among alleles were often represented as networks or bifurcating trees.

### Datasets and polymorphism levels

Simulated datasets encompassed a broad spectrum of polymorphism levels (*A*_N _= 6-22, *G*_N _= 6-42, *H*_O _= 0.26-0.94), and the number of ambiguous genotypes ranged from 2-46 (Table 2). These polymorphism levels were considerably higher than those for each of the genetically-distinct Collembola populations, but at least superficially similar to the mean values obtained for the Collembola data pooled across loci and species (i.e., *S *= 19, *A*_N _= 15, and *G*_N _= 23; Table 2). Most papers included in the literature survey simultaneously analyzed multi-population datasets with PHASE, and reported *A*_N _values were usually within or slightly above the range seen in our empirical datasets. In this context, the present paper should provide a useful framework for understanding impacts of unresolved genotypes and lost alleles on downstream phylogeographic analyses. On the other hand, large differences exist between empirical versus simulated values of *H*_O_, and we recognize that this discrepancy warrants some caution when drawing generalizations from present study. A likely reason for this discrepancy is the presence of geographic substructure within and among Collembola populations. Generally speaking, where geographic substructure exists, rare alleles can occur at locally high frequencies with at least some in homozygous form, rather than at uniformly low frequencies and always as heterozygotes. Ultimately this would reduce heterozygosity, and in particular, may result in fewer ambiguous genotypes that contain rare alleles never before seen in homozygous form.

### PHASE error, unresolved genotypes and lost alleles

PHASE inferences were consistent across replicate runs, indicating that search settings were adequate. Where several alternative solutions for a particular haplotype pair were recovered in the replicate with the best average goodness-of-fit, we used the reconstruction with the highest confidence probability value. If necessary, we randomly selected one of the equally well-support alternatives.

#### Error

**Figure 1:**
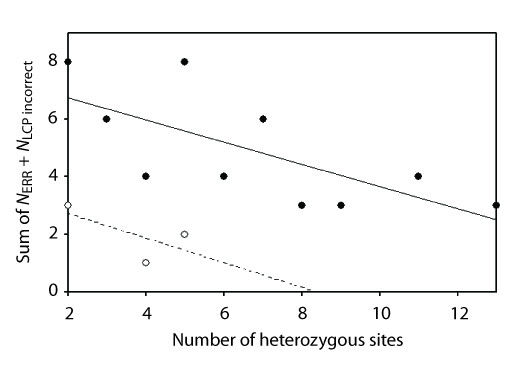
**Relationship between the number of heterozygous sites in an ambiguous genotype (*x*-axis) and haplotype pair reconstruction error (*y*-axis)**. Simulated datasets (solid circles) and empirical datasets (open circles) both showed strong negative correlations (r = -0.692 and r = -0.655, respectively). The plot is identical for the 0.90 and 0.60 PHASE confidence probability thresholds (the latter not shown).

The relationship between PHASE error (*N*_ERR _+ *N*_LCP incorrect_) and the number of heterozygous sites in an ambiguous genotype was negative for simulated and empirical datasets (r = -0.692 and r = -0.655, respectively; Figure 1 solid circles vs. open circles). Under the 0.90 threshold, false positives (*N*_ERR_) were very rare when considering all simulated datasets together (5/1077 ambiguous genotypes = 0.5%), and use of the lower stringency threshold had little impact (9/1077 = 0.8%; Table 3). Similarly, no increase in false positives was seen for the pooled empirical data (3/126 = 2.4% error under both thresholds). For those datasets in which false positives occurred, error rates were as high as 12.5% (*Uc3*), but all others were <10% irrespective of PHASE threshold (Sim06, up to 8%; Sim11, 8.3%; Sim33, 4.9%; *UcEF-1α*, 2.4%). Although only 2-3 of 35 simulated datasets (5.7-8.6%) included at least one false positive, this proportion was higher for the empirical datasets (2/5 = 40% under both thresholds). In all cases, the most prevalent type of mistake was where one haplotype is misidentified and the other is novel (*E*_MIS + NOV_; Table 3).

**Table 3:** Accuracy of PHASE haplotype pair reconstructions.

	Error	0.90 threshold		0.60 threshold	
			
Dataset	category	*90-N*_ERR_	*90-N*_LCP incorrect_	*60-N*_ERR_	*60-N*_LCP incorrect_
Simulated	*E*_COR + NOV_	-	1	-	1
	*E*_COR + MIS_	2	-	2	-
	*E*_MIS + MIS_	-	2	-	2
	*E*_MIS + NOV_	3	41	7	37
	Total	5	44	9	40

Empirical	*E*_COR + NOV_	-	-	-	-
	*E*_COR + MIS_	-	-	-	-
	*E*_MIS + MIS_	-	-	-	-
	*E*_MIS + NOV_	3	3	3	3
	Total	3	3	3	3

False positives (*N*_ERR_) and incorrect reconstructions that had appropriately low confidence probabilities (*N*_LCP incorrect_) are reported for simulated and empirical datasets under two alternative PHASE confidence probability thresholds. Error categories reflect the nature of inference mistakes, and are described in Methods.

#### Unresolved genotypes

**Figure 2:**
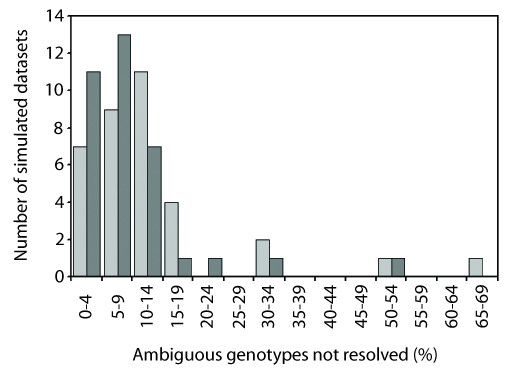
**Frequency distribution of the number of unresolved genotypes (*N*_LCP_) represented as a proportion of the total number of ambiguous genotypes present in each simulated dataset**. Distributions for PHASE confidence probability thresholds 0.90 and 0.60 are shown in pale grey and dark grey, respectively.

Most simulated datasets had some genotypes that were not resolved at or above the specified confidence levels (*N *= 33 and 30 datasets under thresholds 0.90 and 0.60, respectively). Under the 0.90 threshold, the number of these unresolved genotypes (90-*N*_LCP_) per simulated dataset ranged from 0-12 (mean = 3.37). Overall, 62.7% of these unresolved genotypes were nonetheless inferred correctly. Lowering the PHASE threshold to 0.60 led to reductions in the number of unresolved genotypes, but also in the proportion of correctly reconstructed haplotype pairs (60-*N*_LCP _range: 0-7, mean: 2.34, proportion correct: 51.2%). The same general trends were seen in the empirical datasets (90-*N*_LCP _range: 0-7, mean: 3, proportion correct: 80%; 60-*N*_LCP _range: 0-3, mean: 0.80, proportion correct: 25%). When *N*_LCP _was represented as a proportion of the number of ambiguous genotypes (i.e., multi-site heterozygotes) present in each simulated dataset, the frequency distribution for the percentage of ambiguous genotypes that were not resolved under the 0.90 PHASE threshold (mode = 10-14% category; Figure 2, pale grey bars) is slightly off-set to the right compared to that of the 0.60 threshold (mode = 5-9% category; Figure 2, dark grey bars). This indicates that enforcement of a higher-stringency limit on acceptable confidence probability scores generally leads to an increased proportion of unresolved genotypes per dataset.

For the simulated data, regression analyses showed that significant positive relationships exist between each of the four polymorphism measures and *N*_LCP _(Additional file 5). With the exception of *H*_O _(Additional file 5, G-H), the overall strength of each relationship increased considerably as the PHASE threshold was decreased from 0.90 to 0.60, indicating that inclusion of 'marginal' haplotype pairs in the *N*_LCP _category (i.e., those with confidence probability scores of 0.61-0.89) mostly contributes noise. For both thresholds, the strongest predictor variable was *A*_N _(Additional file 5, C-D; 90-*N*_LCP_: slope = 0.264, d.f. = 1,33, *F *= 11.0, *P *= 0.002, *R*^2 ^= 0.25; 60-*N*_LCP_: slope = 0.246, d.f. = 1,33, *F *= 24.7, *P *< 0.001, *R*^2 ^= 0.43). Regressions of the empirical data showed no significant relationships between *N*_LCP _and any of the four polymorphism measures, but these analyses were limited by small sample sizes (*N *= 5 data points). Qualitatively, only one predictor variable (*H*_O_) showed a marked discrepancy between slopes of simulated versus empirical datasets (slope = 7.30 vs. 24.78), but this was limited to the higher PHASE threshold, whereas there was essentially no difference under the lower stringency settings (slope = 5.62 vs. 5.97; Additional file 5). Overall, the simulated datasets generated under panmixia, and the empirical datasets that include considerable substructuring, show similar relationships with polymorphism measures.

**Figure 3:**
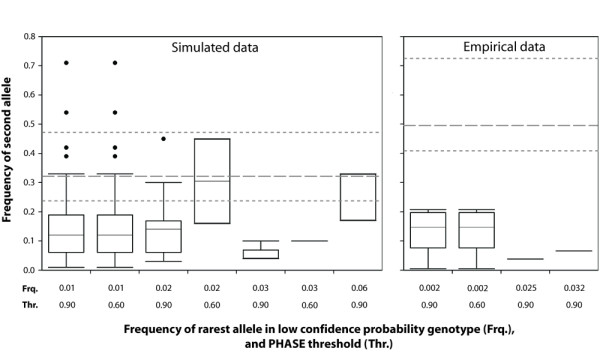
**Genotypic configurations of unresolved genotypes in simulated and empirical datasets**. Population allele frequency values for the more common allele in an unresolved genotype are shown on a continuous scale (*y*-axis), with a separate box plot drawn for each observed value of the rarer allele in an unresolved genotype (*x*-axis). In each plot, the box represents the inner 50% quantile (median marked by a solid black line), and the whiskers represent the upper and lower 25% quantile, excluding outliers (solid black circles). For comparative purposes, the population frequency of the most common allele present in each dataset was used to calculate an overall median and inner 50% quantile (dashed grey lines) for simulated and empirical datasets.

The majority of unresolved genotypes in simulated datasets included at least one singleton allele (70% of 90-*N*_LCP _and 94% of 60-*N*_LCP_). A considerable number of these (19.5% of 90-*N*_LCP _and 20.7% of 60-*N*_LCP_) included a second 'rare' allele (i.e., frequency < 0.05), indicating that genotypic configurations involving two low-frequency alleles are disproportionately represented (Figure 3). Other aspects of *N*_LCP _genotypic configurations also indicate that they are non-random with respect to population allele frequencies. For example, there is usually no overlap between the inner 50% quantile calculated for 'frequency of most common allele in an unresolved genotype' versus that calculated for 'frequency of most common allele in the dataset' (Figure 3 left panel). The two exceptions (i.e., rarest allele frequency = 0.02 and 0.06 under PHASE thresholds 0.90 and 0.60, respectively; Figure 3) both have upper and lower 25% quantiles that do not extend beyond their inner 50% quantile, and so are likely to have been impacted by small sample sizes. If these two tentatively unreliable box plots are ignored, only two meaningful comparisons across PHASE thresholds are possible for the simulated datasets (i.e., 'frequency of rarest allele' categories 0.01 and 0.02). Qualitatively, empirical datasets show the same general patterns relating to genotypic configuration of unresolved genotypes seen in simulated data, and PHASE thresholds do not appear to alter outcomes (Figure 3 right panel).

#### Lost alleles

**Figure 4:**
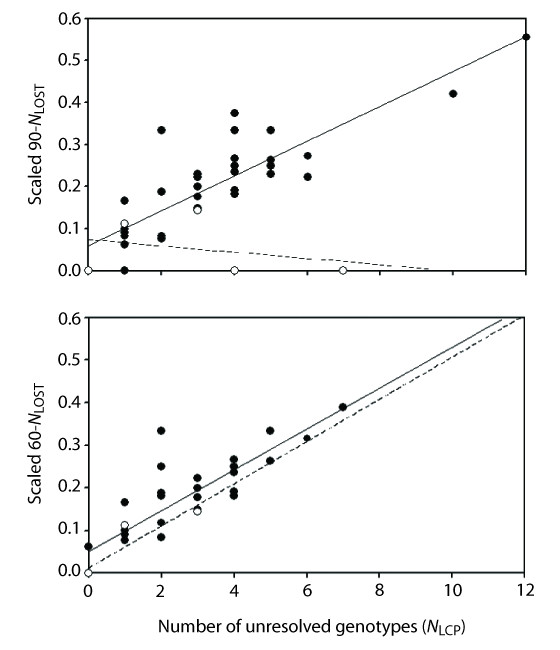
**Relationship between the number of unresolved genotypes (*x*-axis) and reduction in the total number of gene lineages (*y*-axis)**. Top: simulated datasets (solid circles) and empirical datasets (open circles) examined under a PHASE confidence probability threshold of 0.90. Bottom: simulated and empirical datasets examined under the 0.60 threshold. Except for the empirical data under the 0.90 threshold, all regressions were significantly positive (*P *< 0.0001).

The number of lost alleles per simulated dataset ranged from 1-10 or 1-7 (0.90 or 0.60 threshold, respectively; mean = 3 for both). Regression analyses showed a significant positive relationship between *N*_LCP _and reductions in the number of gene lineages in simulated datasets (scaled *N*_LOST_; Figure 4 solid circles). The nature of this relationship was similar for both PHASE thresholds (90-*N*_LCP_: slope = 0.041, d.f. = 1,33, *F *= 92.2, *R*^2 ^= 0.74, *P *<< 0.0001, Figure 4 top; 60-*N*_LCP_: slope = 0.048, d.f. = 1,33, *F *= 92.9, *R*^2 ^= 0.74, *P *<< 0.0001, Figure 4 bottom), indicating that inclusion of 'marginal' haplotype pairs in the 90-*N*_LCP _category contributes to this trend. This systematic loss of phylogenetic diversity with increasing *N*_LCP _was mirrored by the empirical data under the 0.60 threshold, but unexpectedly, not the 0.90 threshold (Figure 4 open circles).

**Figure 5:**
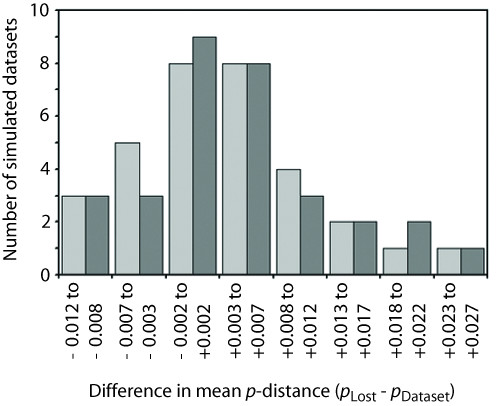
**Frequency distribution of the difference in mean *p*-distance for only those pair-wise comparisons involving lost alleles (*p*_LOST_) and mean from all alleles within a dataset (*p*_DATASET_)**. Distributions for PHASE confidence probability thresholds 0.90 and 0.60 are shown in pale grey and dark grey, respectively.

When considering pairwise sequence divergences among alleles, there are indications that lost alleles tend to be more divergent than retained alleles in simulated datasets. Although distributions of the difference in mean *p*-distance (*p*_LOST _- *p*_DATASET_) under both PHASE thresholds show modal values centered on zero (histogram category -0.002 to + 0.002), the plots are right-skewed (Figure 5). This trend was also seen when summarizing the data using other measures of central tendency and degree of asymmetry (0.90 threshold: *p*_LOST _- *p*_DATASET _mean = 0.0038, skew = 0.520; 0.60 threshold: mean = 0.0044, skew = 0.466). Loss of divergent alleles owing to below-threshold PHASE confidence probability scores of reconstructed haplotype pairs also extends to empirical datasets (*Uc180*, *N*_LOST _= 1, *UcEF-1α*, *N*_LOST _= 3; *p*_LOST _- *p*_DATASET _mean = 0.0060 under both thresholds).

### Standard phylogeographic analyses

**Figure 6:**
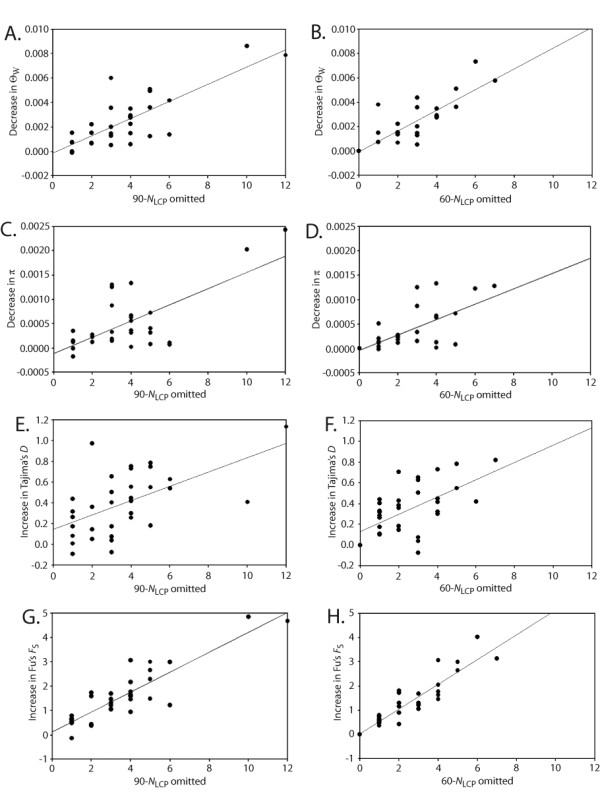
**Relationship between the number of unresolved genotypes omitted from a dataset (*x*-axis) and under- or over-estimation of population genetic parameters commonly used in phylogeographic analyses (*y*-axis)**. A-B, decrease in theta (Θ_W_) under the 0.90 and 0.60 thresholds; C-D, decrease in nucleotide diversity (π) under the 0.90 and 0.60 thresholds; E-F, increase in Tajima's *D *under the 0.90 and 0.60 thresholds; G-H, increase in Fu's *F*_S _under the 0.90 and 0.60 thresholds.

Estimated values of four commonly-used population genetic parameters showed marked directional biases as a consequence of omitting unresolved genotypes from simulated datasets, and the magnitude of these biases was similar for the two PHASE thresholds (Figure 6). The two measures of genetic diversity (Θ_W _and π) were increasingly underestimated as more unresolved genotypes were omitted, whereas the two measures of demographic growth or neutrality (Tajima's *D *and Fu's *F*_S_) were progressively overestimated. Regression analyses confirmed that relationships were significant (all *P*-values < 0.001), with the strongest relationships seen for decreases in Θ_W _(*R*^2 ^= 0.64 and 0.70, Figure 6A-B, respectively) and increases in Fu's *F*_S _(*R*^2 ^= 0.78 and 0.83, Figure 6G-H, respectively). Moreover, paired t-tests showed that parameter values obtained after removing unresolved genotypes differed significantly from those of the corresponding phase-known datasets (all *P*-values < 0.0001 with d.f. = 32; Figure 6A: t = 6.39, Figure 6B: t = 6.67, Figure 6C: t = 4.71, Figure 6D: t = 4.96, Figure 6E: t = -7.47, Figure 6F: t = -8.03, Figure 6G: t = -8.07, Figure 6H: t = -7.62). When considering all simulated datasets together, the maximum downward bias affecting Θ_W _and π were relatively small considering the 'true' mean values estimated from the original phase-known datasets (mean Θ_W _= 0.0160, bias = 0.0086; mean π = 0.0157, bias = 0.0024). However, at the level of individual datasets, reductions in Θ_W _were ≥ 20% of the 'true' value for 9-10 of the 33 simulated datasets with unresolved genotypes. For the pooled simulated data, the two demographic growth or neutrality parameters showed large maximum upward biases compared to the true values (mean *D *= -0.057, bias = 1.135; mean *F*_S _= -0.412, bias = 4.847).

**Figure 7:**
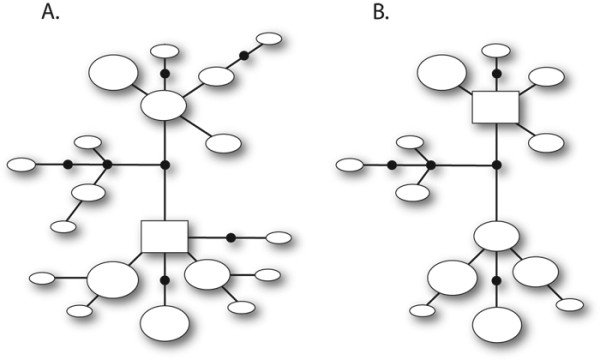
**Statistical parsimony networks constructed for simulated dataset 'Sim21' using TCS **[49]**with the 95% confidence criterion enforced**. A: full dataset (i.e., 100 sequences from 50 diploid genotypes). B: pruned dataset with five unresolved genotypes omitted. Ovals are distinct haplotypes and are drawn proportional to haplotype frequency. Each single line represents one mutational step, and small circles dividing single lines are inferred haplotypes that were not present in the dataset. A rectangle indicates the haplotype with the highest outgroup probability in each network. In this particular case, both the 0.90 and 0.60 PHASE thresholds produced identical outcomes.

In our study, the omission of unresolved genotypes led to changes in the rooting of haplotype networks in three of 35 simulated datasets (8.6%). The example of a root switching error shown in Figure 7 was also seen in a dataset ('Sim26') that produced a more complex set of three disconnected networks. In the latter case, switch errors affected two of these networks. However, changes in the rooting of haplotype networks do not always involve root switching. For example, one of our simulated datasets ('Sim33') produced two disconnected networks, but following removal of unresolved genotypes, these were subsequently split into four (the same outcome resulted under both 90-*N*_LCP _and 60-*N*_LCP _thresholds). This was caused by the loss of a single allele that occupied an important position in one of the original networks-a position that served as a link between otherwise distantly-related alleles (not shown).

## Discussion

### Haplotype reconstruction errors

False positive PHASE inferences, defined here as ambiguous genotypes for which above-threshold haplotype pair reconstructions included errors, were generally very low (Table 3). At the level of individual datasets, rates were usually <10% (with the exception of one empirical dataset, *Uc3*). Considering all simulated datasets together, false positive rates were <1%, and <3% for the pooled empirical data. Haplotype pair reconstruction errors usually involved misidentifying an existing allele coupled with the creation of a novel allele, such that both inferred haplotypes were incorrect (Table 3). The misidentified allele was usually inferred to be the most common allele in the dataset (35-39% or 83% of *E*_MIS + NOV _for simulated or empirical datasets, respectively). Our data also showed a negative correlation between the number of heterozygous sites and PHASE error (c.f. [18] and [23]), and so even two-site heterozygotes can be difficult to reconstruct accurately (Figure 1). However, given the low false positive rates, overestimation of common allele frequencies is unlikely to impact downstream analyses.

Low false positives from PHASE have been reported in several studies based on simulated data and/or well-characterized functional loci in humans [10-18]. The present work, together with two recent papers [23,24], extends these assessments to non-coding anonymous or intronic nDNA from other organisms. The consistently good performance suggests that PHASE is robust to some violations of the underlying neutral coalescent model (e.g., selection, kin clustering, population structure [53]).

### Drivers of uncertainty

Although highly polymorphic nuclear sequence loci often carry considerable phylogeographic signal, they also tend to produce challenging datasets for computational haplotype reconstruction. The number of different alleles (*A*_N_) is a particularly strong predictor of the number of unresolved (*N*_LCP_) genotypes because allele-rich datasets usually contain many rare alleles. Indeed, the presence of a rare allele in an ambiguous genotype is perhaps the single most important determinant of PHASE's ability to confidently and accurately reconstruct haplotype pairs [19]. In populations that have undergone relatively recent and rapid range expansion, coalescent theory predicts an excess of low-frequency haplotypes [28,36,37], and so demographic history may contribute to the number of unresolved genotypes.

Our investigation of the influence of rare alleles on PHASE confidence probabilities indicated that genotypic configurations involving two low-frequency alleles were disproportionately represented in the pool of unresolved genotypes (Figure 3). In out-crossing panmictic populations, these heterozygotes will tend to be very uncommon. However, in hybrid zones, the propensity for rare alleles to reach locally high frequencies or for novel alleles to be found only in individuals of mixed ancestry is well documented [54]. In these cases, the coupling of two otherwise rare alleles in a single diploid genotype may account for a non-negligible proportion of the total dataset. For example, in a study of the *Passerina amoena *(Lazuli bunting) and *P. cyanea *(Indigo bunting) hybrid zone, Carling and Brumfield [52] reported that PHASE was unable to confidently resolve genotypes of as many as 21 individuals per locus. Phylogeographic studies often detect the signals of both range expansion and secondary contact [5,6,55,56]. Accordingly, complex organismal histories may have a compounding effect on the number of unresolved genotypes.

### Impacts of omitting unresolved genotypes

Rare alleles constitute an important component of the molecular signature used to estimate several population genetic parameters, and so omitting genotypes in which rare alleles reside could affect phylogeographic analyses. However, as noted by Edwards and Bensch [27], this has not previously been assessed. We found that systematic biases do exist, and that the absolute number of unresolved genotypes omitted from a dataset is a significant predictor of the magnitude of bias. The parameters Θ_W _and π tend to be underestimated (Figure 6A-D), whereas Fu's *F*_*S *_and Tajima's *D *tend to be overestimated (Figure 6E-H). Even when only 3-4 unresolved genotypes are omitted, parameter estimates can still be quite biased (Figure 6). Although Θ_W _and π were mostly used for descriptive purposes in the studies included in our literature survey, they are increasingly important in phylogeographic hypothesis-testing. For example, estimates of Θ may be used to set effective population size (*N*_*e*_) when modelling alternative vicariance scenarios [4,21,31-33], and it is widely appreciated that fixed parameters such as *N*_*e *_can have a large impact on the outcome of such tests. The parameter Θ can also be important when ranking alternative models under an information-theoretic framework for phylogeographic inference [57]. Similarly, the combination of Tajima's *D *and π has been identified as particularly powerful when testing simultaneous vicariance under an approximate Bayesian computation inference framework [58].

Tests for distinguishing population growth from size constancy often use information from the frequency of distribution of DNA substitutions or haplotypes, where an excess of singletons is indicative of expansion [59]. In the absence of selection and intra-locus recombination, significantly negative values of Tajima's *D *and Fu's *F*_*S *_are consistent with population growth. Indeed, many species have experienced rapid population expansions since the Last Glacial Maximum. In these cases, current practices of omitting unresolved genotypes should have greater impacts on analyses than those seen from simulations performed under a model of constant population size. However, even under the best-case scenario represented in simulations, our results indicate a strong upward bias in these two statistics when unresolved genotypes are omitted from a dataset (Figure 6E-H). In a highly cited paper, Ramos-Onsins and Rozas [59] reported on the superiority of Fu's *F*_*S *_to detecting population growth, and this statistic is now widely used in phylogeographic analyses. The marked overestimation of *F*_*S *_reported in the present paper could ultimately mask the signature of expansion. In a comparative phylogeographic context, understanding species' demographic histories is critical for assessing the degree to which co-distributed taxa have responded to past landscape-level events in concert [5,6,60,61]. Given the increasing number of studies that include re-analysis of datasets generated by other research groups [62,63], it may become necessary to make a clear distinction between computationally- versus experimentally-phased nDNA sequence datasets.

Some comparative phylogeographic analyses focus on the topology and branch lengths of estimated gene trees. Here we have shown that omitting genotypes with low confidence probability scores usually leads to reductions in two components of phylogenetic diversity-the number of gene lineages and pairwise sequence divergences among them (Figure 4, 5). Rare alleles can be particularly difficult to resolve (Figure 3), and so some loss of distinct gene lineages is expected. However, we also detected an unexpected bias towards loss of divergent alleles (c.f. the average *p*-distance among all alleles in the dataset). This can alter estimated root probabilities in intraspecific haplotype networks (Figure 7). The implications for downstream network-based analyses (e.g., Nested Clade Phylogeographic Analysis [64]) is an area of research that demands further study, but is beyond the scope of the present paper. The systematic loss of rare alleles could also potentially impact outcomes of molecular dating methods that require removal of short branches [65], or tests of topological congruence between taxa [66]. For these reasons, some caution is warranted even when performing phylogeny-based phylogeographic analyses with computationally-phased datasets.

### Mitigation of observed biases and other sources of error

We have found SSCP to be efficient for physically isolating alleles from diploid PCR products [38,41], but the utility of cloning or allele-specific PCR has also been demonstrated (Table 1). Regardless of which approach is considered most feasible, we reiterate the point made by Huang *et al*. [23] that the effort invested in experimental haplotype determination can be minimized by targeting genotypes that remain unresolved following computational approaches. Indeed, it may not be necessary to experimentally phase all unresolved genotypes given that biases in the four population parameter estimates examined here were always quite low when unresolved genotypes accounted for ≤ 2% of the total dataset under the 0.90 PHASE threshold (Figure 6). Notably, lowering the PHASE threshold to 0.60 often reduces the number of unresolved genotypes with little or no increase in false positives (Table 3).

The potential for some genotyping error to arise when scoring heterozygous sites from directly-sequenced diploid PCR products is well-documented. For example, base composition bias can contribute to highly asymmetric signal intensities [9], chain termination sequencing chemistry may cause certain nucleotides to produce small peaks compared to other bases at the same heterozygous position [67,68], and the sequencing primers themselves can have a substantial effect on accuracy [69]. Furthermore, variable sites in close proximity to the 5' or 3' ends of an alignment may be difficult to score accurately [24], although it is not clear if this is a general phenomenon. Problems may also arise when one allele amplifies in PCR with low efficiency relative to another allele [70]. Taken together, if inconsistencies are seen between forward and reverse sequence chromatograms for the same diploid template, it would be prudent to experimentally validate these genotypes. Indeed, Bos *et al*.'s [53] recommendation for ground-truthing a sub-sample of the haplotypes inferred by PHASE is well justified.

## Conclusions

In contrast to Harrigan *et al*. [24], we have not been able to escape the conclusion that a combination of experimental and computational approaches for resolving phase of segregating sites in phylogeographic applications is essential. We have shown that the current practice of omitting unresolved genotypes (i.e., those that cannot be resolved with high confidence using computational approaches implemented in PHASE) introduces systematic bias into estimates of important population genetic parameters. As expected, these biases have their most pronounced effects on summary statistics that draw on the signal embedded in the number of rare alleles (e.g., tests of neutrality or population growth). Fortunately, with targeted application of laboratory procedures that enable unambiguous phase determination via physical isolation of alleles from diploid PCR products (e.g., cloning, allele-specific PCR, SSCP), relatively little investment of time and effort is needed to overcome potential biases. This notion that the 'best' strategy involves a duality of approaches represents a recurring theme in phylogeography [71-73].

## Authors' contributions

RCG conceived the study, performed the analyses, and drafted the paper. PS and RJD contributed ideas to the study design and interpretation of results, and revised drafts of the paper. All authors read and approved the final manuscript.

## Supplementary Material

Additional file 1 **Increase over time in the use of PHASE in empirical studies relating to phylogeography, speciation or hybridization**. Figure is based on the 60 articles included in our literature survey (see Table 1 in the main text). All of these studies focus on non-primate animals and used PHASE to reconstruct haplotypes from directly sequenced non-coding nuclear loci. Click here for file

Additional file 2 **Frequency distribution of the relationship between number of segregating sites (S) and number of different alleles (A_N_) in the 500 simulated datasets from which 35 (solid circles) were arbitrarily selected for further analysis using PHASE**. Figure shows that none of the 35 datasets are atypical (i.e., outliers), and so the results presented in the main text are free from bias relating to the dataset selection procedure. Click here for file

Additional file 3 **Correlation coefficients between the four measures of dataset polymorphism**. In this figure, values were calculated from the pooled empirical datasets (above diagonal), and pooled simulated datasets (below diagonal). S, number of segregating sites; A_N_, number of different alleles; G_N_, number of different genotypes; H_O_, observed heterozygosity. Click here for file

Additional file 4 **Supplementary references**. List of 60 papers from 18 journals included in the literature survey of empirical studies that used PHASE for haplotype reconstruction (see Table 1 of the main text). Click here for file

Additional file 5 **Relationship between alternative measures of dataset polymorphism (x-axis) and the number of unresolved genotypes (y-axis)**. Simulated and empirical datasets are represented by solid circles and open circles, respectively. A-B, number of segregating sites (S) under the 0.90 and 0.60 thresholds; C-D, number of different alleles (A_N_) under the 0.90 and 0.60 thresholds; E-F, number of different genotypes (G_N_) under the 0.90 and 0.60 thresholds; G-H, observed heterozygosity (H_O_) under the 0.90 and 0.60 thresholds. All regressions were significantly positive (P < 0.05) for simulated data, but not for the empirical data. Click here for file
