# Supplementary material for: Nuclear gene phylogeography using PHASE: dealing with unresolved genotypes, lost alleles, and systematic bias in parameter estimation
Source: BMC Evol Biol. 2010 Apr 30;10:118. doi: 10.1186/1471-2148-10-118 (PMC2880299; doi:10.1186/1471-2148-10-118)
Supplement: Additional file 1 — Increase over time in the use of PHASE in empirical studies relating to phylogeography, speciation or hybridization. Figure is based on the 60 articles included in our literature survey (see Table 1 in the main text). All of these studies focus on non-primate animals and used PHASE to reconstruct haplotypes from directly sequenced non-coding nuclear loci. [file 1471-2148-10-118-S1.PDF]

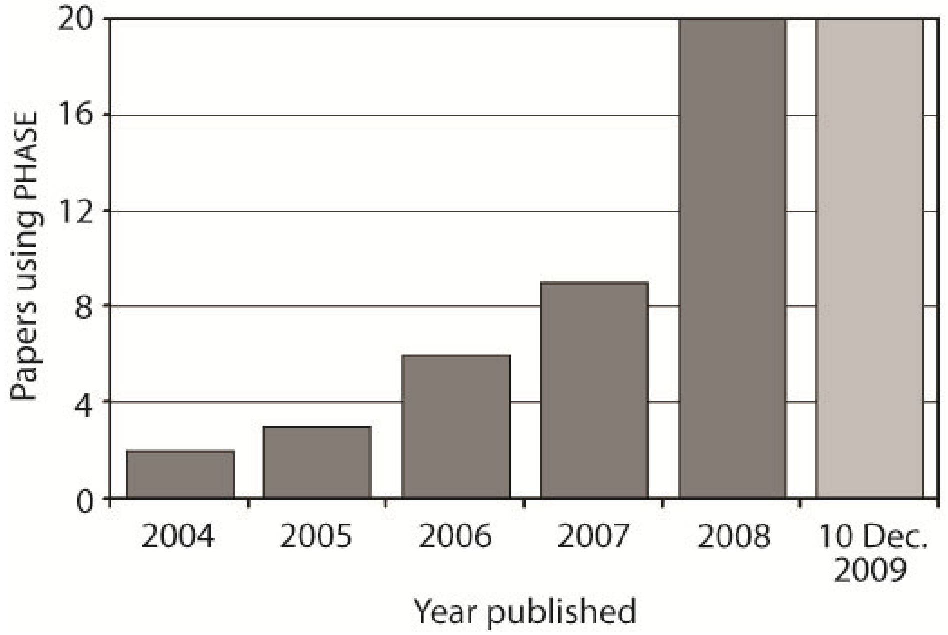

Additional file 1. ***Increase over time in the use of PHASE in empirical studies relating to phylogeography, speciation or hybridization***. *Figure is based on the 46 articles included in our literature survey (see Table 1 in the main text). All of these studies focus on non-primate animals and used PHASE to reconstruct haplotypes from directly sequenced non-coding nuclear loci.*
