# Supplementary material for: Nuclear gene phylogeography using PHASE: dealing with unresolved genotypes, lost alleles, and systematic bias in parameter estimation
Source: BMC Evol Biol. 2010 Apr 30;10:118. doi: 10.1186/1471-2148-10-118 (PMC2880299; doi:10.1186/1471-2148-10-118)
Supplement: Additional file 2 — Frequency distribution of the relationship between number of segregating sites (S) and number of different alleles (AN) in the 500 simulated datasets from which 35 (solid circles) were arbitrarily selected for further analysis using PHASE. Figure shows that none of the 35 datasets are atypical (i.e., outliers), and so the results presented in the main text are free from bias relating to the dataset selection procedure. [file 1471-2148-10-118-S2.PDF]

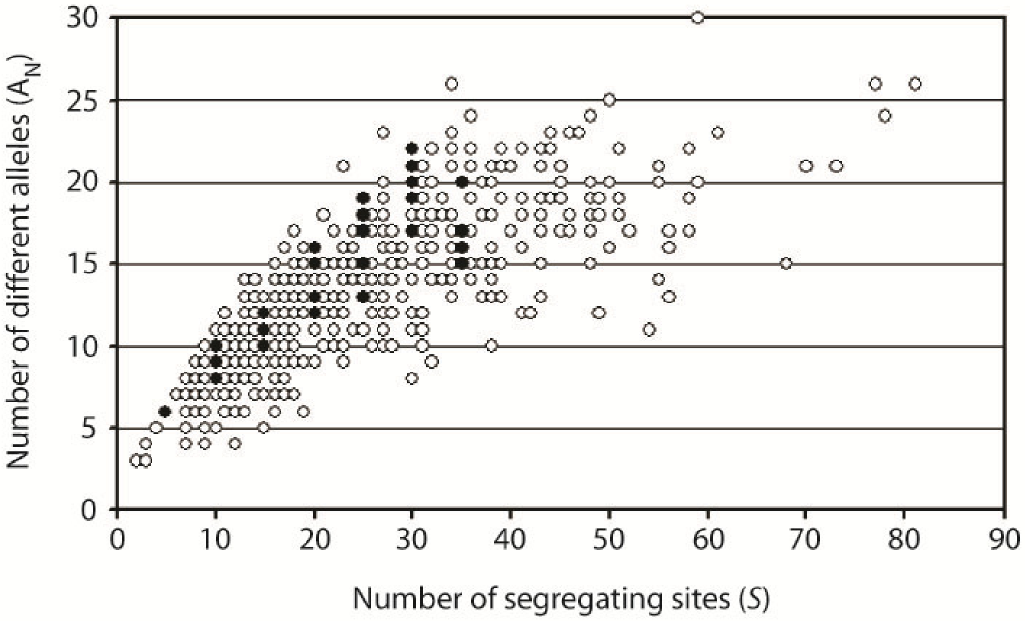

Additional file 2. ***Frequency distribution of the relationship between number of segregating sites (S) and number of different alleles (A_N) in the 500 simulated datasets from which 35 (solid circles) were arbitrarily selected for further analysis using PHASE.*** *Figure shows that none of the 35 datasets are atypical (i.e., outliers), and so the results presented in the main text are free from bias relating to the dataset selection procedure.*
