# Supplementary material for: Nuclear gene phylogeography using PHASE: dealing with unresolved genotypes, lost alleles, and systematic bias in parameter estimation
Source: BMC Evol Biol. 2010 Apr 30;10:118. doi: 10.1186/1471-2148-10-118 (PMC2880299; doi:10.1186/1471-2148-10-118)
Supplement: Additional file 3 — Correlation coefficients between the four measures of dataset polymorphism. In this figure, values were calculated from the pooled empirical datasets (above diagonal), and pooled simulated datasets (below diagonal). S, number of segregating sites; AN, number of different alleles; GN, number of different genotypes; HO, observed heterozygosity. [file 1471-2148-10-118-S3.PDF]

Additional file 3. ***Correlation coefficients between the four measures of dataset polymorphism.*** *In this figure, values were calculated from the pooled empirical datasets (above diagonal), and pooled simulated datasets (below diagonal). S, number of segregating sites;* A_N*, number of different alleles;* G_N*, number of different genotypes;* H_O*, observed heterozygosity.*

	Correlation coefficient			
	S	A_N	G_N	H_O
S	–	0.809	0.922	0.773
A_N	0.906	–	0.971	0.955
G_N	0.860	0.940	–	0.945
H_O	0.625	0.662	0.758	–
