# Supplementary material for: Nuclear gene phylogeography using PHASE: dealing with unresolved genotypes, lost alleles, and systematic bias in parameter estimation
Source: BMC Evol Biol. 2010 Apr 30;10:118. doi: 10.1186/1471-2148-10-118 (PMC2880299; doi:10.1186/1471-2148-10-118)
Supplement: Additional file 4 — Supplementary references. List of 60 papers from 18 journals included in the literature survey of empirical studies that used PHASE for haplotype reconstruction (see Table 1 of the main text). [file 1471-2148-10-118-S4.PDF]

Additional file 4. ***Supplementary references.*** *List of 60 papers from 18 journals included in the literature survey of empirical studies that used* PHASE *for haplotype reconstruction (see Table 1 of the main text).*

Birds.

Balakrishnan CN, Edwards SV (2009) Nucleotide variation, linkage disequilibrium and founder–facilitated speciation in wild populations of the zebra finch (*Taeniopygia guttata*). *Genetics*, **181**, 645–660.

Balakrishnan CN, Sefc KM, Sorenson MD (2009) Incomplete reproductive isolation following host shift in brood parasitic indigobirds. *Proceedings of the Royal Society B*, **276**, 219–228.

Berlin S, Quintela M, Höglund J (2008) A multilocus assay reveals high nucleotide diversity and limited differentiation among Scandinavian willow grouse (*Lagopus lagopus*). *BMC Genetics*, **9**, 89.

Bowie RCK, Fjeldsa J, Kiure J (2009) Multilocus molecular DNA variation in Winifred's Warbler *Scepomycter winifredae* suggests cryptic speciation and the existence of a threatened species in the Rubeho–Ukaguru Mountains of Tanzania. *Ibis*, **151**, 709–719.

Carling MD, Brumfield RT (2008) Haldane's rule in an avian system: using cline theory and divergence population genetics to test for differential introgression of mitochondrial, autosomal, and sex–linked loci across the *Passerina* bunting hybrid zone. *Evolution*, **62**, 2600–2615.

Carling MD, Brumfield RT (2009) Speciation in *Passerina* buntings: introgression patterns of sex–linked loci identify a candidate gene region for reproductive isolation. *Molecular Ecology*, **18**, 834–847.

Cheviron ZA, Brumfield RT (2009) Migration–selection balance and local adaptation of mitochondrial haplotypes in Rufous–Collared Sparrows (*Zonotrichia capensis*) along an elevational gradient. *Evolution*, **63**, 1593–1605.

Lee JY, Edwards SV (2008) Divergence across Australia's Carpentarian Barrier: statistical phylogeography of the red–backed fairy wren (*Malurus melanocephalus*). *Evolution*, **62**, 3117–3134.

McCracken KG, Bulgarella M, Johnson KP *et al*. (2009) Gene flow in the face of countervailing selection: adaptation to high–altitude hypoxia in the βA hemoglobin subunit of yellow–billed pintails in the Andes. *Molecular Biology and Evolution*, **26**, 815–827.

Mettler RD, Spellman GM (2009) A hybrid zone revisited: Molecular and morphological analysis of the maintenance, movement, and evolution of a Great Plains avian (Cardinalidae: *Pheucticus*) hybrid zone. *Molecular Ecology*, **18**, 3256–3267.

Peck DR, Bancroft WJ, Congdon BC (2008) Morphological and molecular variation within an ocean basin in wedge–tailed shearwaters (*Puffinus pacificus*). *Marine Biology*, **153**, 1113–1125.

Peters JL, Zhuravlev Y, Fefelov I, Humphries EM, Omland KE (2008) Multilocus phylogeography of a Holarctic duck: colonization of North America from Eurasia by gadwall (*Anas strepera*). *Evolution*, **62**, 1469–1483.

Peters JL, Zhuravlev Y, Fefelov I, Logie A, Omland KE (2007) Nuclear loci and

coalescent methods support ancient hybridization as a cause of mitochondrial paraphyly between gadwall and falcated duck (*Anas* spp.). *Evolution*, **61**, 1992–2006.
Sonsthagen SA, Talbot SL, McCracken KG (2007) Genetic characterization of common eiders breeding in the Yukin–Kuskokwim Delta, Alaska. *Condor*, **109**, 878–893.
Sonsthagen SA, Talbot SL, Lanctot RB, Scribner KT, McCracken KG (2009) Hierarchical spatial genetic structure of Common Eiders (*Somateria mollissima*) breeding along a migratory corridor. *The Auk*, **126**, 744–754.

Herpetofauna.

Bos DH, Gopurenko D, Williams RN, DeWoody JA (2008) Inferring population history and demography using microsatellites, mitochondrial DNA, and major histocompatibility complex (MHC) genes. *Evolution*, **62**, 1458–1468.
Carnaval AC, Bates JM (2007) Amphibian DNA shows marked genetic structure and tracks Pleistocene climate change in northeastern Brazil. *Evolution*, **61**, 2942–2957.
Carstens BC, Stoute HN, Reid NM (2009) An information–theoretical approach to phylogeography. *Molecular Ecology*, **18**, 4270–4282.
Fitzpatrick SW, Brasileiro CA, Haddad CFB, Zamudio KR (2009) Geographical variation in genetic structure of an Atlantic Coastal Forest frog reveals regional differences in habitat stability. *Molecular Ecology*, **18**, 2877–2896.
Fonseca MM, Brito JC, Paulo OS, Carretero MA, Harris DJ (2009) Systematic and phylogeographical assessment of the *Acanthodactylus erythrurus* group (Reptilia: Lacertidae) based on phylogenetic analyses of mitochondrial and nuclear DNA. *Molecular Phylogenetics and Evolution*, **51**, 131–142.
Gifford ME, Larson A (2008) *In situ* genetic differentiation in a Hispaniolan lizard (*Ameiva chrysolaema*): A multilocus perspective. *Molecular Phylogenetics and Evolution*, **49**, 277–291.
Guarnizo CE, Amézquita A, Bermingham E (2009) The relative roles of vicariance versus elevational gradients in the genetic differentiation of the high Andean tree frog, *Dendropsophus labialis*. *Molecular Phylogenetics and Evolution*, **50**, 84–92.
Pinho C, Harris DJ, Ferrand N (2008) Non–equilibrium estimates of gene flow inferred from nuclear genealogies suggest that Iberian and North African wall lizards (*Podarcis* spp.) are an assemblage of incipient species. *BMC Evolutionary Biology*, **8**, 63.
Rosenblum EB, Hickerson MJ, Moritz C (2007) A multilocus perspective on colonization accompanied by selection and gene flow. *Evolution*, **61**, 2971–2985.
Vogel LS, Johnson SG (2008) Estimation of hybridization and introgression frequency in toads (Genus: *Bufo*) using DNA sequence variation at mitochondrial and nuclear loci. *Journal of Herpetology*, **42**, 61–75.
Weisrock DW, Shaffer HB, Storz BL, Storz SR, Voss SR (2006) Multiple nuclear gene sequences identify phylogenetic species boundaries in the rapidly radiating clade of Mexican ambystomatid salamanders. *Molecular Ecology*, **15**, 2489–2503.
Zarza E, Reynoso VH, Emerson BC (2008) Diversification in the northern neotropics: mitochondrial and nuclear DNA phylogeography of the iguana *Ctenosaura pectinata* and related species. *Molecular Ecology*, **17**, 3259–3275.

Mammals.

Carneiro M, Ferrand N, Nachman MW (2009) Recombination and speciation: Loci near centromeres are more differentiated than loci near telomeres between subspecies of the European rabbit (*Oryctolagus cuniculus*). *Genetics*, **181**, 593–606.

Conroy CJ, Neuwald JL (2008) Phylogeographic study of the California vole, *Microtus californicus*. *Journal of Mammalogy*, **89**, 755–767.

Geraldes A, Basset P, Gibson B *et al*. (2008) Inferring the history of speciation in house mice from autosomal, X–linked, Y–linked and mitochondrial genes. *Molecular Ecology*, **17**, 5349–5363.

Geraldes A, Carneiro M, Delibes–Mateos M *et al*. (2008) Reduced introgression of the Y chromosome between subspecies of the European rabbit (*Oryctolagus cuniculus*) in the Iberian Peninsula. *Molecular Ecology*, **17**, 4489–4499.

Good JM, Hird S, Reid N *et al*. (2008) Ancient hybridization and mitochondrial capture between two species of chipmunks. *Molecular Ecology*, **17**, 1313–1327.

Harlin–Cognato AD, Markowitz T, Würsig B, Honeycutt RL (2007) Multi–locus phylogeography of the dusky dolphin (*Lagenorhynchus obscurus*): passive dispersal via the west–wind drift or response to prey species and climate change? *BMC Evolutionary Biology*, **7**, 131.

Hellborg L, Gündüz İ, Jaarola M (2005) Analysis of sex–linked sequences supports a new mammal species in Europe. *Molecular Ecology*, **14**, 2025–2031.

Stevison LS, Kohn MH (2009) Divergence population genetic analysis of hybridization between rhesus and cynomolgus macaques. *Molecular Ecology*, **18**, 2457–2475.

Tchaicka L, Eizirik E, de Oliveira TG, Cândido JF Jr., Freitas RO (2007) Phylogeography and population history of the crab–eating fox (*Cerdocyon thous*). *Molecular Ecology*, **16**, 819–838.

Väli Ü, Einarsson A, Waits L, Ellegren H (2008) To what extent do microsatellite markers reflect genome–wide genetic diversity in natural populations? *Molecular Ecology*, **17**, 3808–3817.

Fish.

Bigg GR, Cunningham CW, Ottersen G *et al*. (2008) Ice–age survival of Atlantic cod: agreement between palaeoecology models and genetics. *Proceedings of the Royal Society B*, **275**, 163–173.

Gaffney PM, Rupnow J, Domeier ML (2007) Genetic similarity of disjunct populations of the giant sea bass *Stereolepis gigas*. *Journal of Fish Biology*, **70**, 111–124.

Janko K, Lecointre G, DeVries A *et al*. (2007) Did glacial advances during the Pleistocene influence differently the demographic histories of benthic and pelagic Antarctic shelf fishes? – Inferences from intraspecific mitochondrial and nuclear DNA sequence diversity. *BMC Evolutionary Biology*, **7**, 220.

Kuhn KL, Gaffney PM (2006) Preliminary assessment of population structure in the mackerel icefish (*Champsocephalus gunnari*). *Polar Biology*, **29**, 927–935.

Ryynänen HJ, Primmer CR (2004) Distribution of genetic variation in the growth hormone 1 gene in Atlantic salmon (*Salmo salar*) populations from Europe and North America. *Molecular Ecology*, **13**, 3857–3869.

Scott CH, Cashner M, Grossman GD, Wares JP (2009) An awkward introduction:

Phylogeography of *Notropis lutipinnis* in its 'native' range and the Little Tennessee River. *Ecology of Freshwater Fish*, **18**, 538–549.
Taylor MS, Hellberg ME (2006) Comparative phylogeography in a genus of coral reef fishes: biogeographic and genetic concordance in the Caribbean. *Molecular Ecology*, **15**, 695–707.
von der Heyden S, Prochazka K, Bowie RC (2008) Significant population structure and asymmetric gene flow patterns amidst expanding populations of *Clinus cottoides* (Perciformes, Clinidae): application of molecular data to marine conservation planning in South Africa. *Molecular Ecology*, **17**, 4812–4826.

Invertebrates.

Calderón I, Giribet G, Turon X (2008) Two markers and one history: phylogeography of the edible common sea urchin *Paracentrotus lividus* in the Lusitanian region. *Marine Biology*, **154**, 137–151.
Dyer KA, Jaenike J (2005) Evolutionary dynamics of a spatially structured host–parasite association: *Drosophila innubila* and male–killing *Wolbachia*. *Evolution*, **59**, 1518–1528.
Evans LM, Allan GJ, Shuster SM, Woolbright SA, Whitham TG (2008) Tree hybridization and genotypic variation drive cryptic speciation of a specialist mite herbivore. *Evolution*, **62**, 3027–3040.
Hurt C, Anker A, Knowlton N (2009) A multilocus test of simultaneous divergence across the Isthmus of Panama using snapping shrimp in the genus *Alpheus*. *Evolution*, **63**, 514–530.
Johnson SG (2006) Geographic ranges, population structure, and ages of sexual and parthenogenetic snail lineages. *Evolution*, **60**, 1417–1426.
Johnson SB, Young CR, Jones WJ, Warén A, Vrijenhoek RC (2006) Migration, isolation, and speciation of hydrothermal vent limpets (Gastropoda; Lepetodrilidae) across the Blanco Transform Fault. *Biological Bulletin*, **210**, 140–157.
Kawakami T, Butlin RK, Adams M *et al.* (2007) Differential gene flow of mitochondrial and nuclear DNA markers among chromosomal races of Australian morabine grasshoppers (*Vandiemenella*, *vatica* species group). *Molecular Ecology*, **16**, 5044–5056.
Kawakami T, Butlin RK, Adams M, Saint KM *et al.* (2009) Re–examination of a proposed case of stasipatric speciation: Phylogeography of the Australian morabine grasshoppers (*Vandiemenella viatica* species group). *Molecular Ecology*, **18**, 3429–3442.
Llopart A, Lachaise D, Coyne JA (2005) Multilocus analysis of introgression between two sympatric sister species of *Drosophila*: *Drosophila yakuba* and *D. santomea*. *Genetics*, **171**, 197–210.
Mathews LM (2006) Cryptic biodiversity and phylogeographical patterns in a snapping shrimp species complex. *Molecular Ecology*, **15**, 4049–4063.
Mathews LM, Adams L, Anderson E *et al.* (2008) Genetic and morphological evidence for substantial hidden biodiversity in a freshwater crayfish species complex. *Molecular Phylogenetics and Evolution*, **48**, 126–135.
Nunes F, Norris RD, Knowlton N (2009) Implications of isolation and low genetic

diversity in peripheral populations of an amphi–Atlantic coral. *Molecular Ecology*, **18**, 4283–4297.

Papadopoulou A, Anastasiou I, Keskin B, Vogler AP (2009) Comparative phylogeography of tenebrionid beetles in the Aegean archipelago: The effect of dispersal ability and habitat preference. *Molecular Ecology*, **18**, 2503–2517.

Smith CI, Godsoe WKW, Tank S, Yoder JB, Pellmyr O (2008) Distinguishing coevolution from covicariance in an obligate pollination mutualism: asynchronous divergence in Joshua tree and its pollinators. *Evolution*, **62**, 2676–2687.

Sotka EE, Wares JP, Barth JA, Grosberg RK, Palumbi SR (2004) Strong genetic clines and geographical variation in gene flow in the rocky intertidal barnacle *Balanus glandula*. *Molecular Ecology*, **13**, 2143–2156.
