# Supplementary material for: Nuclear gene phylogeography using PHASE: dealing with unresolved genotypes, lost alleles, and systematic bias in parameter estimation
Source: BMC Evol Biol. 2010 Apr 30;10:118. doi: 10.1186/1471-2148-10-118 (PMC2880299; doi:10.1186/1471-2148-10-118)
Supplement: Additional file 5 — Relationship between alternative measures of dataset polymorphism (x-axis) and the number of unresolved genotypes (y-axis). Simulated and empirical datasets are represented by solid circles and open circles, respectively. A-B, number of segregating sites (S) under the 0.90 and 0.60 thresholds; C-D, number of different alleles (AN) under the 0.90 and 0.60 thresholds; E-F, number of different genotypes (GN) under the 0.90 and 0.60 thresholds; G-H, observed heterozygosity (HO) under the 0.90 and 0.60 thresholds. All regressions were significantly positive (P < 0.05) for simulated data, but not for the empirical data. [file 1471-2148-10-118-S5.PDF]

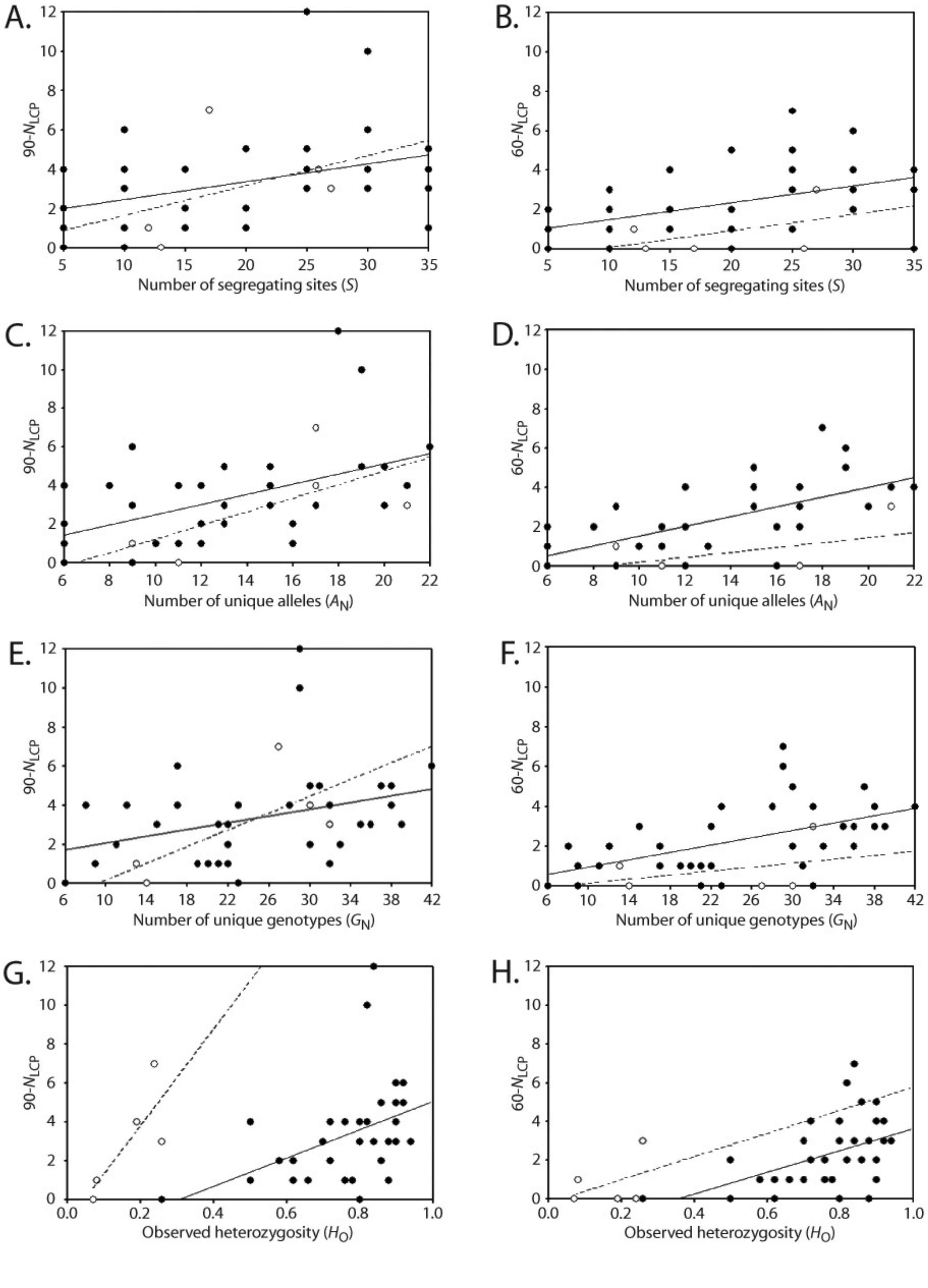

Additional file 5. ***Relationship between alternative measures of dataset polymorphism (x-axis) and the number of unresolved genotypes (y-axis).*** *Simulated and empirical datasets are represented by solid circles and open circles, respectively. A–B, number of segregating sites (S) under the 0.90 and 0.60 thresholds; C–D, number of different alleles (A_N) under the 0.90 and 0.60 thresholds; E–F, number of different genotypes (G_N) under the 0.90 and 0.60 thresholds; G–H, observed heterozygosity (H_O) under the 0.90 and 0.60 thresholds. All regressions were significantly positive ($P < 0.05$) for simulated data, but not for empirical data.*
